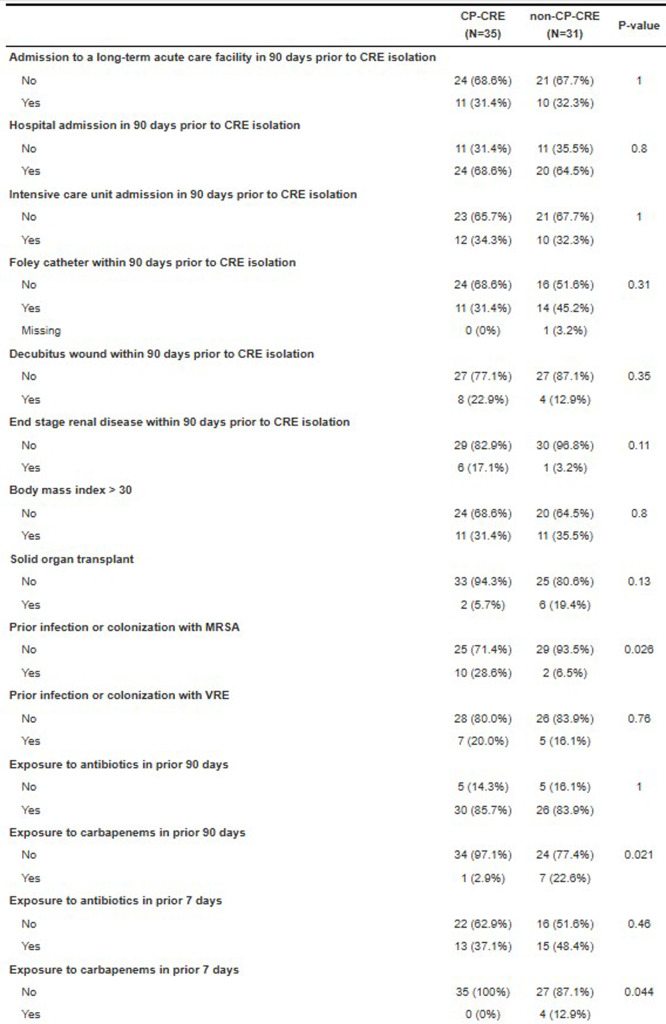# 301 Increased Contamination of Blood Cultures Collected via New Peripheral IV Start vs Venipuncture

**DOI:** 10.1017/ash.2026.10656

**Published:** 2026-06-23

**Authors:** Scott Curry, Robert Sean Tyler, Savannah Lee, Shelly Maguire, Cassandra Salgado

**Affiliations:** 1 Medical University of South Carolina

## Abstract

**Background:** Within-hospital transmission has been reported with carbapenem-resistant Enterobacterales (CRE) infections. At our medical center, much of the CRE submitted for carbapenemase surveillance is non-carbapenemase producing (non-CP-CRE). We explored risk factors for CP-CRE vs non-CP-CRE infections in a cohort in our center in which both CP-CRE and CRE overall are rare to explore the hypothesis that horizontal transmission is more likely with CP-CRE. **Methods:** All patients with meropenem-resistant Enterobacterales isolated at MUSC March 2022 through February 2025 that were submitted to the state public health lab for modified carbapenem inactivation method (mCIM) testing and reflex metallobetalactamase gene PCR (CARBA-R, Cepheid) were included. Patients with positive mCIM were defined as carbapenemase-producing CRE (CP-CRE). A subset of CP-CRE patients from January 2024-February 2025 was compared to patients with non-carbapenemase-producing CRE over the same period and underwent chart review regarding antimicrobial exposures, past infection with other MDROs, past healthcare facility exposure, and other clinical features. **Results:** Over the study period 170 total CRE isolates were forwarded, of which 83 (48%) were confirmed CP-CRE, of which KPC (68, 82%) and NDM (15, 18%), and oxa-48-like (1, 1%) were the mechanisms detected. Klebsiella pneumoniae (54/83, 65%) and Enterobacter spp. (35/87, 40%) were the most prevalent CP-CRE and non-CP-CRE species, respectively. From January 2024 – February 2025, 35 CP-CRE and 31 non-CP-CRE-infected patients were reviewed. CP-CRE was associated with resistance to several non-beta-lactam antibiotics whereas non-CP-CRE was significantly associated with prior exposure to carbapenem-class antibiotics in the prior 90 days (23% vs 3%, P = 0.021). No other significant differences in the two groups were noted apart from prior MRSA infection and solid organ transplant status associated with non-CP-CRE (Table). **Conclusion:** CP-CRE is associated with more significant multi-class resistance and much less associated than non-CP-CRE with prior carbapenem exposure, suggesting that horizontal transmission may play a more important role in CP-CRE infections whereas in-patient evolution of CRE may play a larger role in non-CP-CRE infections.

**Figure:**
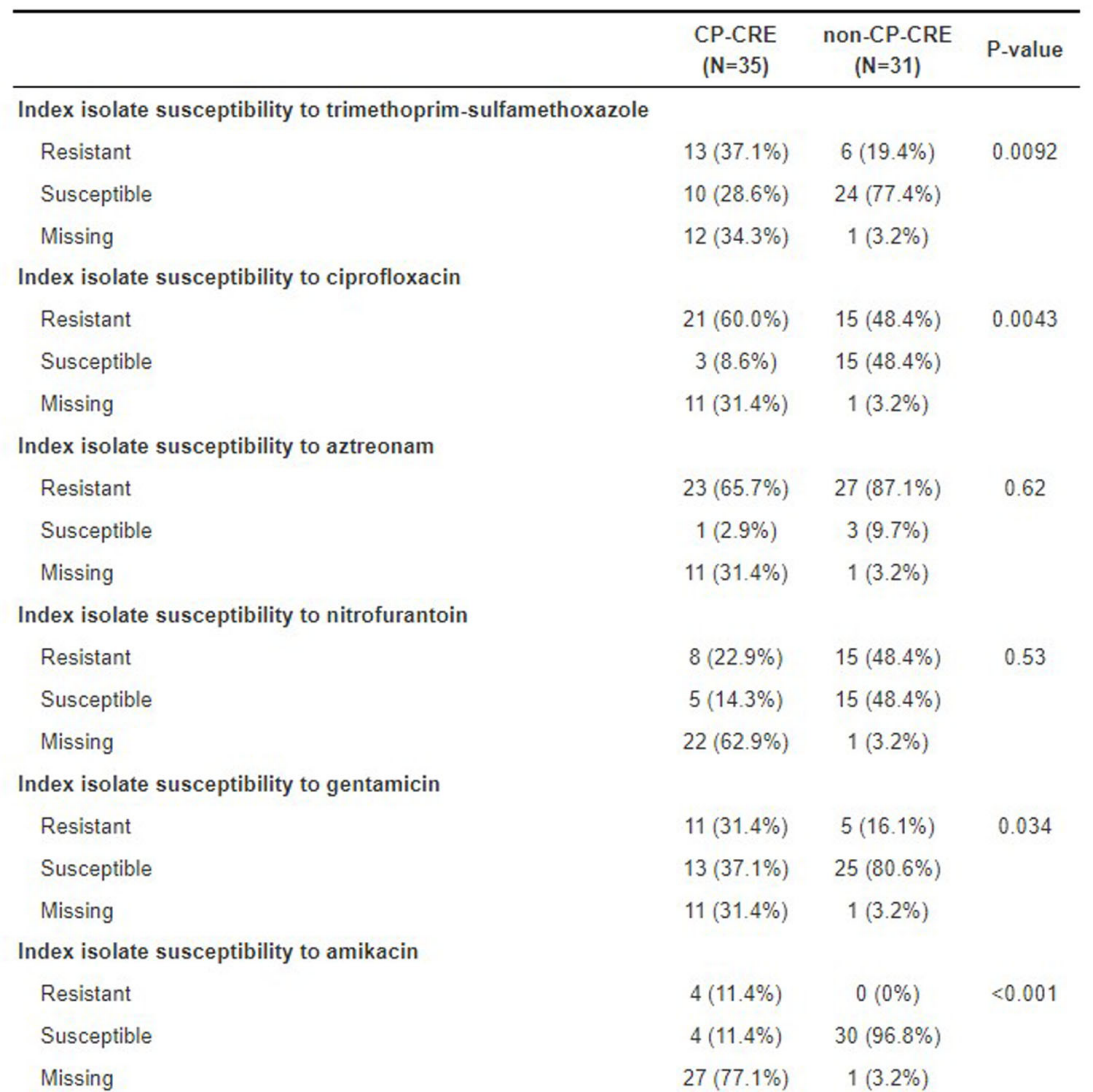

**Figure:**